# Supplementary figures and images for: Global sociodemographic, clinical, and epidemiological profiling of patients with mycetoma: A systematic review
Source: PLoS Negl Trop Dis. 2025 Aug 14;19(8):e0013217. doi: 10.1371/journal.pntd.0013217 (PMC12617881; doi:10.1371/journal.pntd.0013217)

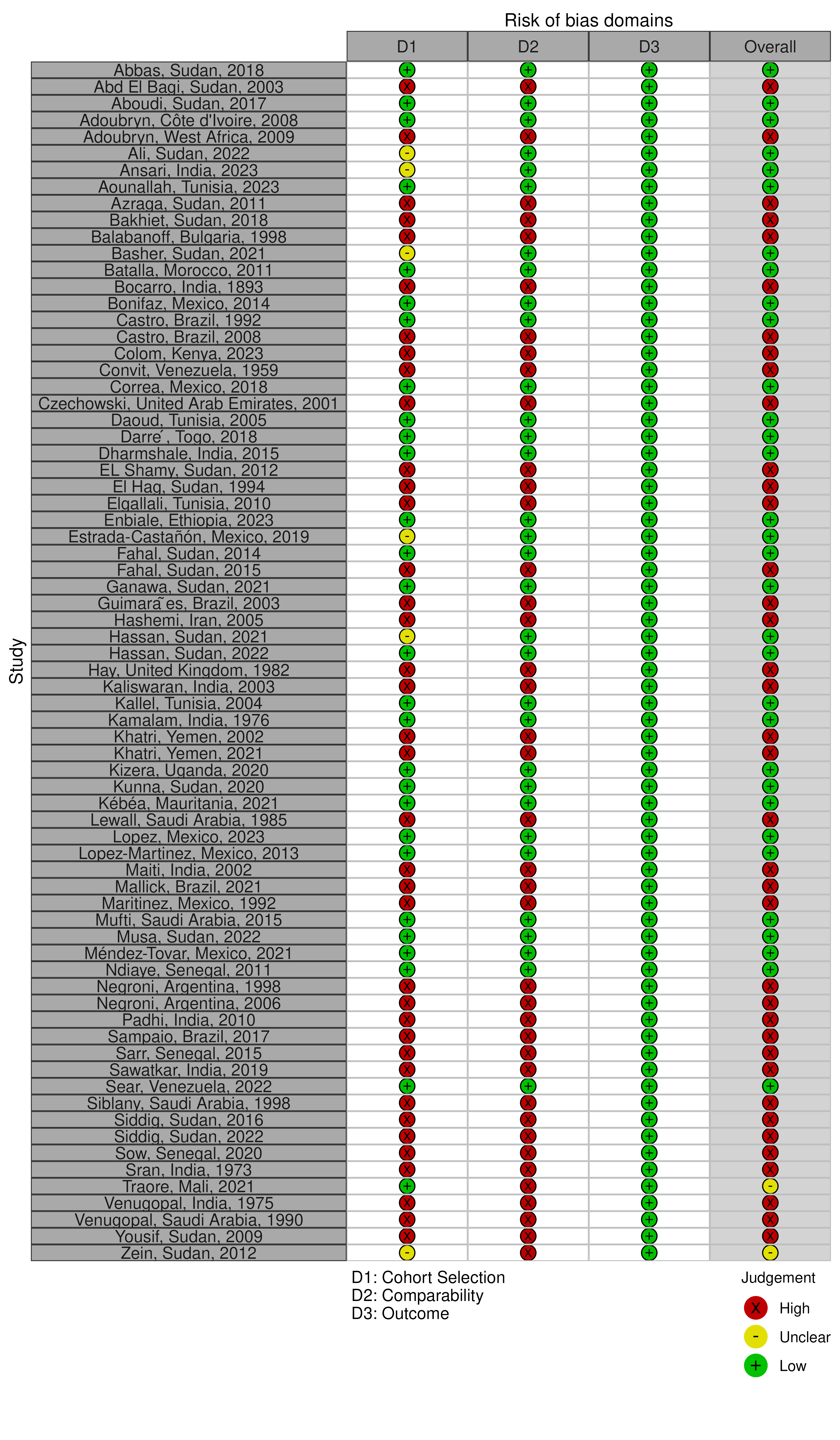

Supplement: S1 Fig — (PNG) [file pntd.0013217.s006.png]
