# Supplementary material for: Global sociodemographic, clinical, and epidemiological profiling of patients with mycetoma: A systematic review
Source: PLoS Negl Trop Dis. 2025 Aug 14;19(8):e0013217. doi: 10.1371/journal.pntd.0013217 (PMC12617881; doi:10.1371/journal.pntd.0013217)
Supplement: Attachment — Submitted filename: Response to Reviewers.docx [file pntd.0013217.s010.docx]

**Manuscript Title:** *Global Sociodemographic, Clinical, and Epidemiological Profiling of Patients with Mycetoma: A Systematic Review*

 **Journal:** *PLOS Neglected Tropical Diseases*

Dear Editors,

We thank you and the reviewers for your thoughtful feedback and the opportunity to revise our manuscript. We have carefully addressed all reviewer and editorial comments as outlined below:

### **Reviewer and Editorial Comments Addressed: (highlighted in blue)**

1. A brief explanation of study heterogeneity was added to the *Methods* section.
2. A paragraph on public health implications was included in the *Discussion*.
3. A clarification regarding outdated taxonomic names of causative agents was added to the *Discussion.*
4. Figures were processed through PACE and updated.
5. A complete list of Supporting Information legends was added after the references list.
6. The Data Availability statement is updated and will be provided during the submission.
7. Table S4 data was added to the manuscript in the *Results* section
8. Table 3 was added, describing actinomycetoma vs. eumycetoma cases, although the data was sufficiently discussed in the manuscript and the figures.

### **Additional Modifications by the MRC Team: (highlighted in blue)**

While responding to the editor and reviewer comments, we reproofed the manuscript one more time and made a few edits:

- The manuscript was re-edited for language and grammar.
- The referencing style was updated to PLOS Vancouver.
- Table layouts were refined for clarity and consistency.

We appreciate your time and consideration and hope the revised manuscript meets the journal’s expectations.

Kind regards,
