# Supplementary material for: Global sociodemographic, clinical, and epidemiological profiling of patients with mycetoma: A systematic review
Source: PLoS Negl Trop Dis. 2025 Aug 14;19(8):e0013217. doi: 10.1371/journal.pntd.0013217 (PMC12617881; doi:10.1371/journal.pntd.0013217)
Supplement: Attachment — Submitted filename: Reviewer Comments.pdf [file pntd.0013217.s008.pdf]

Reviewer Comments:

Many thanks for inviting me to review manuscript PNTD-D-25-00408 for PLOS Neglected Tropical Diseases. Please find my comments below:

*The title and Abstract is otherwise well written and covers all the essential aspects.

*The introduction provides comprehensive and relevant background information for the study.

*The Methods Well-structured and PRISMA-aligned. You need to mention how data heterogeneity or missing data were handled, for transparency per PRISMA-2020.

* Results you could briefly mention **data limitations**, such as missing variables or uneven geographical data coverage, which often affects neglected disease reviews.

*The discussion findings correlate appropriately with the results. They are relevant, well-referenced, and contextualized within the existing literature. The authors also correctly highlight limitations and propose future directions (e.g., data standardization and global surveillance).

* No further modifications/suggestions.
